# Exploring the Epicarp Potential from *Acrocomia aculeata* Fruits: Chemical Analysis, Antioxidant and Antimicrobial Activities

**DOI:** 10.3390/antiox14020181

**Published:** 2025-02-04

**Authors:** Fabiane da Conceição Vieira Santos, Gabriel Rocha Martins, Sandra Regina da Silva Luiz, Isadora de Araújo Oliveira, Leandro Pereira da Silva, Antonio Jorge Ribeiro da Silva, Marcos Dias Pereira, Rosana Conrado Lopes, Celuta Sales Alviano, Daniela Sales Alviano Moreno

**Affiliations:** 1 Graduate Program in Food Science (PPGCAL), Institute of Chemistry (IQ), Federal University of Rio de Janeiro (UFRJ), Cidade Universitária, Rio de Janeiro 21941-909, RJ, Brazil; fabiiane@ufrj.br; 2 Department of Pharmacy, School of Pharmaceutical Sciences, University of São Paulo, São Paulo 05508-000, SP, Brazil; gabrielrmartins@usp.br; 3 Graduate Program in Science (PPG-Micro), Department of General Microbiology, Institute of Microbiology Paulo de Góes (IMPG), Federal University of Rio de Janeiro (UFRJ), Cidade Universitária, Rio de Janeiro 21941-902, RJ, Brazil; sandraregipn2514@micro.ufrj.br; 4 Institute of Biophysics Carlos Chagas Filho, Centro de Espectrometria de Massas de Biomoléculas (CEMBIO), Federal University of Rio de Janeiro (UFRJ), Cidade Universitária, Rio de Janeiro 21941-902, RJ, Brazil; isadora@biof.ufrj.br; 5 Graduate Program in Plant Biotechnology and Bioprocesses (PBV), Federal University of Rio de Janeiro (UFRJ), Cidade Universitária, Rio de Janeiro 21941-902, RJ, Brazil; leandropereiira@ufrj.br; 6 Natural Products Research Institute (IPPN), Federal University of Rio de Janeiro (UFRJ), Cidade Universitária, Rio de Janeiro 21941-902, RJ, Brazil; ajorge@ippn.ufrj.br; 7 Department of Biochemistry, Institute of Chemistry (IQ), Federal University of Rio de Janeiro (UFRJ), Cidade Universitária, Rio de Janeiro 21941-909, RJ, Brazil; marcosdp@iq.ufrj.br; 8 Department of Botany, Institute of Biology (IB), Federal University of Rio de Janeiro (UFRJ), Cidade Universitária, Rio de Janeiro 21941-909, RJ, Brazil; rosana@biologia.ufrj.br; 9 Department of General Microbiology, Institute of Microbiology Paulo de Góes (IMPG), Federal University of Rio de Janeiro (UFRJ), Cidade Universitária, Rio de Janeiro 21941-902, RJ, Brazil; alviano@micro.ufrj.br

**Keywords:** macaúba epicarp, agricultural by-products, stilbenes, antioxidant activity, antimicrobial activity, mass spectrometry imaging, *Saccharomyces cerevisiae*, *Galleria mellonella*

## Abstract

The interest in new sources of bioactive compounds has been driven by the search for natural antioxidants capable of attenuating the toxicity of reactive oxygen species, as well as the emergence of pathogens resistant to antimicrobials. In this sense, we explored the potential of the macaúba epicarp. Compounds such as piceatannol, 3,4,5,3′,5′-penta-hydroxy-trans-stilbene (PHS), and in lower amounts, resveratrol were identified in extracts through techniques such as medium-pressure liquid chromatography, HPLC-MS, and imaging mass spectrometry (IMS), which confirmed the exclusive localization of PHS and piceatannol in the outer epicarp. Extraction with aqueous acetone (Me_2_CO:H_2_O) and its EtOAC fraction showed the highest yields of stilbenes and, moreover, it efficiently increased the tolerance of *Saccharomyces cerevisiae* to oxidative stress. Additionally, the Me_2_CO:H_2_O extract presented antibacterial and anti-cryptococcal activity, with piceatannol and resveratrol increasing survival rates of *Galleria mellonella* subjected to fungal infection. In silico ADMET (absorption, distribution, metabolism, excretion and toxicity) analysis indicates low toxicity for piceatannol, PHS, and resveratrol, in addition to pharmacokinetic parameters that allow their use. These findings indicate the use of macaúba epicarp as a source of bioactive compounds valuable for the food, cosmetic, and pharmaceutical industries.

## 1. Introduction

*Acrocomia aculeata* (Jacq.) Lodd. ex Mart, commonly known as macaúba, is an oleaginous palm tree native to the tropical and subtropical regions of the Americas [1]. In Brazil, it is primarily found in the Southeast and Midwest regions [2], and is recognized as a promising alternative source of oil for the production of biodiesel and biokerosene due to its high production potential and oil quality [3,4]. This species begins its production between 4 and 6 years of age and remains productive for over 100 years [3]. A single plant can yield between 2 and 8 bunches, with each bunch containing 250 to 500 fruits [1,3]. The estimated productivity is significant, ranging from 4 to 6 tons of oil per hectare and 24 to 40 tons of fruits per hectare per year [1,3,5].

Its fruit is typically spherical, with an average diameter of 2.5 to 5 cm, featuring a smooth shell (epicarp) that readily breaks when ripe. An edible yellowish and fibrous pulp (mesocarp) firmly surrounds the endocarp, which is hard and dense with a blackish wall containing one or even two oleaginous kernels (endosperm) coated by a thin brown integument. The pulp and kernel oils are the most commercially valuable products derived from these fruits. However, the pulp oil primarily contains oleic acid (63–65%) as the main component. Meanwhile, the oil extracted from the kernel is high in lauric acid (38–45%) [3]. The processing of these fruits generates by-products with great potential for reuse [1]. The epicarp (are inedible), represents 19–24% of the fruit’s mass [6,7,8,9], and remains an underexplored source of bioactive compounds, with only a few studies addressing its chemical composition [10]. Currently, its use has been proposed in the production of biogas [11], of charcoal [12], briquettes [13,14], and production of bio-oil from the pyrolysis process [15].

To fill this gap, exploratory techniques are essential for creating a comprehensive chemical profile and evaluating its bioactivity. Recent studies demonstrate that fruit peels and seeds are valuable sources of bioactive compounds that can be converted into high-value products [16,17,18,19]. Therefore, in this study, we explored the potential of macaúba’s epicarp, an underutilized residue, as a source of bioactive compounds. We identified and quantified stilbenes through the integration of techniques such as imaging mass spectrometry (IMS) that allows for the direct visualization of the spatial distribution of compounds of plant tissue [17,20], and high-performance liquid chromatography coupled with mass spectrometry (HPLC-MS) that enables the quantification and structural elucidation of the plant extracts [21]. Additionally, we evaluated their antioxidant and antimicrobial potential, and we conducted an in silico ADMET prediction for the isolated stilbenes, aiming to highlight the potential of macaúba epicarp as a new source of bioactive compounds.

## 2. Materials and Methods

### 2.1. Standards and Chemicals

Acetonitrile, methanol, ethanol, acetone, n-hexane, toluene, and ethyl acetate of HPLC grade were purchased from Tedia (Rio de Janeiro, Brazil). Formic acid (98%), brain heart infusion (BHI) agar, potassium chloride, D-glucose, peptone, and ascorbic acid were purchased from Merck (Darmstadt, Germany). Resveratrol (3,4′,5-trihydroxy-*trans*-stilbene), piceatannol (3,3′,4,5′-tetrahydroxy-*trans*-stilbene), hydrogen peroxide, resazurin, dimethyl sulfoxide (DMSO), 2,2-diphenyl-1-picrylhydrazyl (DPPH), ciprofloxacin, amphotericin B (AMB), RPMI, MOPS, D-sorbitol, yeast extract, gelatin, carboxymethyl cellulose (CMC), 9-aminoacridine, sodium citrate, and sodium formate were purchased from Sigma-Aldrich (St. Louis, MO, USA). Mueller Hinton broth, Bacto agar, and Sabouraud Dextrose agar were purchased from Becton, Dickinson and Company (Sparks, MD, USA).

### 2.2. Plant Material

Macaúba fruit samples were collected in the morning period in the municipality of Guapimirim, Rio de Janeiro, Brazil (22°38′12.5″ S 42°58′58.3 W), between November and December 2018 and November 2019. Fruits were gathered from three different trees to ensure a representative sample. Dra. Rosana Conrado Lopes (Institute of Biology, Federal University of Rio de Janeiro, Cidade Universitária, RJ, Brazil) authenticated the species and a voucher specimen was deposited at the Herbarium of the Institute of Biology (UFRJ) under the registration number RFA 40.607, and the permission to access genetic heritage was registered in the Sistema Nacional de Gestão do Patrimônio Genético e do Conhecimento Tradicional Associado (SisGen) under the registration number AD192F1.

### 2.3. Processing Plant Material and Extraction Procedures

The collected fruits were cleaned and peeled. All the gathered epicarp was dried in an oven at 50 °C for 24 h, crushed in an analytical mill (IKA, Model: A11; Campinas, SP, Brazil), and standardized by sieving to 35 mesh (425 µm ABNT; BERTEL Indústria Metalúrgica Ltda Caieiras, SP, Brazil). The resulting epicarp powder (827 g) was processed as a single, homogenized sample, packaged, and stored at room temperature for further extractions. The extractions were performed in triplicate using 1.00 g of sample and 10 mL of water (H_2_O), ethanol (EtOH), 60% (*v*/*v*) ethanol in water (EtOH:H_2_O) or 60% (*v*/*v*) acetone in water (Me_2_CO:H_2_O) in a solid/liquid ratio of 1:10 (*m*/*v*), for 10 min, at 25 °C, assisted by an ultrasonic bath (Branson 2510 Ultrasonic Cleaner, Danbury, CT, USA). Then, the extracts were filtered, the solvent evaporated in a rotavapor under reduced pressure, and, afterward, lyophilized. All samples were stocked dry and resuspended in a proper solvent before use.

The antioxidant activity by the DPPH method and antimicrobial activity were evaluated in the crude extracts to select the extract with the best bioactivity profile. After these preliminary tests, the extract Me_2_CO:H_2_O was solubilized in water with the assistance of ultrasound, and then subjected to liquid−liquid partition with ethyl acetate (EtOAc) (1:1). The EtOAc fraction was evaporated in a rotavapor under reduced pressure, and the aqueous fraction was lyophilized.

### 2.4. Purification of the Active Fraction by Medium-Pressure Liquid Chromatography (MPLC)

The EtOAc fraction was purified using an MPLC system (Biotage Isolera, Uppsala, Sweden) combined with the UV module, using a SNAP Ultra silica cartridge (16 mm × 150 mm; Biotage, Uppsala, Sweden). The mobile phase was a binary gradient of toluene (A) and acetone (B). The elution profile was 0–1 min 10% B; 1–11 min 10–86% B; 11–16 min 86% B. The flow rate was 75 mL/min, the detector wavelength was 280 and 321 nm, and the injection volume was 15 mL (2.8 mg/mL). Eluent systems were chosen based on tests previously performed by thin-layer chromatography (TLC). 

The collected fractions were analyzed by thin chromatography on silica gel 60 F254 plates (Merck), with acetone/toluene/formic acid (3:3:0.1, *v*/*v*/*v*) as mobile phase. Immediately after development, the plate was dried with a cold air stream, analyzed under UV light (365 nm), and regrouped by the similarity of the chromatographic profile, giving rise to subfraction 01 (fractions 12–-13) and subfraction 02 (fractions 16–17).

### 2.5. Macaúba Crude Extracts’ Phenolic Profile and Fraction Characterization by High-Performance Liquid Chromatography Tandem Mass Spectrometry (HPLC-MS)

The crude extracts and fractions were analyzed by a Nexera X2 liquid chromatograph (Shimadzu, Chiyoda-ku, TYO, Japan) system connected to a Maxis Impact spectrometer (Bruker Daltonics, Bremen, Germany), with ESI-QTOF configuration. The samples were injected at 2 mg/mL (10 μL injection volume) into a 2.1 mm × 150 mm, 3 μm particle ODS-Hypersil C18 reverse phase column (Thermo Fisher, Waltham, MA, USA) equipped with a 4 mm × 3 mm C18 pre-column (Phenomenex, Torrance, CA, USA), maintained at 35 °C. Elution was performed at 0.3 mL/min in a gradient of mobile phases of water (A) and acetonitrile (B), both containing 0.1% formic acid, as follows: 0 min 5% B, 2 min 5% B, 30 min 15% B, 40 min 30% B, 41 min 90% B, 52 min 90% B, 53 min 5% B, and 60 min 5% B. Spectra were acquired in negative mode in the *m*/*z* range 30 to 1200 at 1.5 Hz in data-dependent acquisition (DDA) mode with fragmentation of the 2 most intense ions, with the *m*/*z* range 200 to 300 of preferred ions. The electrospray source parameters were set as follows: capillary voltage 5000 V, nebulizer gas pressure 4 Bar, drying gas flow rate 8 L/min, source temperature 200 °C. Calibration was performed at each analysis with a 100 μM sodium formate solution infusion in water/acetonitrile 1:1 (*v*/*v*). Data were analyzed using Data Analysis 5.1 (Bruker Daltonics), QuantAnalysis 5.1 (Bruker Daltonics, Germany), and GraphPad Prism 8 (GraphPad Software, Boston, MA, USA).

The UPLC-MS analyses of the MPLC subfractions were conducted using an UltiMate 3000 UHPLC system (Thermo Scientific Dionex, Waltham, MA, USA), equipped with a quaternary gradient pump, solvent degasser, diode array detector (DAD), autosampler, and a column temperature manager. This system was coupled to an LCQ Fleet mass spectrometer (Thermo Fisher Scientific, Waltham, MA, USA). Chromatographic separation was achieved with an Acquity UPLC BEH C18 column (2.1 mm × 100 mm, 1.7 μm, Waters, Milford, MA, USA) at a flow rate of 0.30 mL/min. The column temperature was maintained at 35 °C. The mobile phase consisted of (A) water with 0.1% (*v*/*v*) formic acid and (B) acetonitrile with 0.1% (*v*/*v*) formic acid The following gradient was applied: 0 min 5% B, 2 min 5% B, 30 min 15% B, 40 min 30% B, 41 min 90% B, 52 min 90% B, 53 min 5% B, and 60 min 5% B. The mass spectrometer, equipped with an electrospray ionization (ESI) source, operated in negative-ion mode. High-purity nitrogen (N_2_) was used as both sheath gas (35 arbitrary units) and auxiliary gas (10 arbitrary units), while helium (He) was employed as the collision gas. The source parameters were configured as follows: source voltage of 5.5 kV, capillary voltage of −37 V, tube lens voltage of −101.07 V, and capillary temperature of 350 °C. Mass spectra were acquired in full-scan mode, covering a mass-to-charge ratio (*m*/*z*) range of 50–500, and data processing was performed using Xcalibur^TM^ 2.2 software (Thermo Fisher Scientific, Waltham, MA, USA). For fragmentation studies, a data-dependent acquisition (DDA) method was employed. The scan triggered the fragmentation of the most intense peak from MS1, with a minimum intensity threshold of 200 counts. Collision-induced dissociation (CID) was performed with a normalized collision energy of 35%, and the precursor ion isolation width was set to *m*/*z* 2.0.

### 2.6. Quantification of Stilbenes

The quantification of piceatannol and resveratrol in samples of the crude extracts and fractions was performed by LC-MS (Shimadzu, Japan). The parameters and elution profile were conducted as described in topic 2.5. The detection was carried out by Maxis Impact (ESI-QTOF) mass spectrometer (Bruker Daltonics), operating in negative mode. The quantification was performed by analyzing calibration curves of a mixture of samples added with 0.625–20 µM (resveratrol) and 1.25–40 µM (piceatannol) of commercial standards of these molecules. Data were analyzed using QuantAnalysis 5.1 (Bruker Daltonics, Germany) and GraphPad Prism 8.

### 2.7. Imaging Mass Spectrometry

The macaúba fruits (6 fruits) were kept at −20 °C until the date of preparation. For cutting, the fruits were kept at room temperature for 7 h, and sections tangential to the endocarp, containing epicarp and mesocarp, were obtained using a microtome blade (Leica 818, Nussloch, Germany). The fruit portions were embedded in a solution of 10% gelatin and 5% carboxymethyl cellulose (CMC), frozen in dry ice, and kept at −80 °C. Sections of 20 μm of the embedded material were made in a cryostat (Leica CM1860-UV, Leica Biosystems, Nussloch, Germany) at −15 °C and adhered to the glass slide using double-sided copper tape (3M, Two Harbors, MA, USA). Images of the slide were acquired with a scanner (HP Photosmart, Palo Alto, CA, USA) at 4800 dpi resolution. The deposition of 9-aminoacridine (9AA) was performed using an adapted system consisting of an APCI-type ionization source needle (APCI Apollo II, Bruker Daltonics, Germany) connected to a syringe pump and a nitrogen gas source coupled to the XY printer arm (AxiDraw V3; Evil Mad Scientist, Sunnyvale, CA, USA) to ensure homogeneous dispersion across the entire surface of the slide [22]. Thus, the matrix solution (10 mg/mL of 9AA in a methanol/water solution 80:20 (*v*/*v*)) was infused at 800 μL/h and nebulized with an N_2_ flow at 12.5 psi for 30 min. The spectra were acquired on a Solarix XR 7T mass spectrometer (Bruker Daltonics, Germany) with MALDI-FT-ICR configuration, in negative mode at 2 MW (resolution of 99,000 at *m*/*z* 400) in the previously calibrated range of *m*/*z* 100 to 1000, but using the quadrupole for isolation of ions between *m*/*z* 190 and 270 to increase sensitivity on the analytes of interest. The laser power was adjusted to 70%, and each spectrum was acquired from 200 laser shots with a lateral resolution of 50 μm. The data were processed and analyzed in the FlexImaging software version 5.0 (Bruker Daltonics, Germany).

### 2.8. Measurement of Antioxidant Activity of Macaúba’s Fruit Epicarp Extracts

DPPH antioxidant assay was performed as described previously [23], adapted for microdilution. Stock solutions of bark extracts (3.5 mg/mL) or standards (1.4 mg/mL) were diluted in ethanol to final concentrations of 250–1.9 µg/L (extracts) and 100–0.7 µg/L (standards) in 96-well plates (Kasvi; Pinhais, PR, Brazil). Then, 50 µL of a DPPH solution prepared in ethanol (EtOH) at 0.3 mM was added to the wells containing 125 µL of the diluted sample. The absorbance was measured at 490 nm after 30 min of reaction at 25 °C. To avoid the interference of colors, bark extract diluted solution at each concentration tested (125 µL) plus ethanol (50 µL) was used as blank, except for the analysis of standards (175 µL of EtOH as blank). DPPH solution (50 µL) plus EtOH (125 µL) was used as negative control. The antioxidant activity standards of ascorbic acid, resveratrol, and piceatannol were positive controls. The tests were performed in triplicate and the absorbance values were converted into the percentage antioxidant activity (AA), according to the following equation:

AA% = 100 − {[(Abs sample − Abs blank) × 100]/Abs control}(1)

The final results were expressed as EC50 (the amount of sample necessary to decrease the absorbance of DPPH by 50%) by linear regression analysis.

### 2.9. Evaluation of the Protective Effect Against Reactive Oxygen Species (ROS)

A wild-type strain of *Saccharomyces cerevisiae*, BY4741 (*MATa*; *his3∆1*; *leu2∆0*; *met15∆0*; *ura3∆0*), was used to evaluate the antioxidant activity of the extract Me_2_CO:H_2_O and its EtOAc fraction. Stocks of this strain were maintained on a solid YPD 2% medium (1% yeast extract, 2% glucose, 2% peptone, and 2% agar) under appropriate conditions. For all experiments, cells were grown up to the middle of the exponential phase (0.8–1.0 mg dry weight/mL) in liquid 2% YPD medium, using an orbital shaker at 28 °C and 160 rpm, with a 5:1 ratio of flask volume/medium [24,25]. Next, for cytotoxicity analysis, approximately 1 mg/mL of cells were reinoculated in fresh medium and fresh medium containing 100 µg/mL of the crude extract or fraction for 24 h at 28 °C/under agitation at 160 rpm. Cell growth was determined at different time intervals by measuring absorbance at 570 nm, and the values were converted into the cell mass.

To analyze the antioxidant activity, approximately 1 mg/mL of cells were directly stressed (1 mM H_2_O_2_ for 1 h at 28 °C/160 rpm), or pretreated with 10, 20, and 100 µg/mL of the extract Me_2_CO:H_2_O or EtOAc fraction for 1h at 28 °C and 160 rpm. Immediately after the adaptive treatment, the cells were harvested by centrifugation (3.000 rpm/3 min/25 °C), washed twice with sterile water, resuspended in the original culture medium, and then subjected to oxidative stress. As a control, part of the culture was kept at 28 °C (cells non-treated and non-stressed). After adequate dilution, cell viability was analyzed by plating in triplicate in solid 2% YPD medium. The plates were incubated at 28 °C for 72 h, and colonies were counted. Tolerance was expressed as a percentage of survival [24,25].

### 2.10. Microorganisms and Growth Conditions

The antibacterial and antifungal activities were evaluated against the Gram-negative bacteria *Salmonella enterica* serovar Typhimurium (ATCC 14028); Gram-positive bacteria *Bacillus subtilis*, *Staphylococcus aureus* (ATCC 6538), and methicillin-resistant *Staphylococcus aureus* (MRSA BMB 9393)—a clinical isolate obtained from Hospital Universitário Clementino Fraga Filho (HUCFF/UFRJ); the yeasts *Candida albicans* serotype B (ATCC 36802) and *Cryptococcus neoformans* serotype A (T_1_-444)—clinical isolate obtained from Universidade Federal de São Paulo, Brazil (UNIFESP—SP); *Cryptococcus neoformans* H99 serotype A (ATCC 208821)—provided by D.Sc. Arturo Casadevall Johns Hopkins; and *Cryptococcus neoformans* serotype D (ATCC 24067).

The bacterial and yeast strain stocks were maintained in medium BHI agar and Sabouraud Dextrose agar, respectively, at 4 °C. For the experiments, the microorganisms were grown in BHI agar (for bacteria) for 24 h at 35 °C or in Sabouraud for 24 h at 37 °C (for *Candida*) and 48 h at 37 °C (for *Cryptococcus*)

### 2.11. Evaluation of Minimal Inhibitory Concentration (MIC)

The antimicrobial potential was determined using the microdilution broth method as described by the Clinical Laboratory Standard Institute (CLSI) M7-A6 for bacteria and M27-A2 for yeast [26,27]. Ten microliter stock solutions of epicarp extracts (20 mg/mL) or stilbene standards (5 mg/mL) or antimicrobial standards (1 mg/mL) were diluted to different concentrations in a 96-well plate. Then, after serial dilutions of the samples in triplicate, wells were inoculated with 10 µL of bacterial suspension (5 × 10^6^ CFU/mL) in Mueller–Hinton medium or 100 µL of fungal suspension (5 × 10^3^ CFU/mL) in RPMI-MOPS pH 7.2 medium. The negative and positive controls comprised pure and inoculated growth medium (non-treated cells), respectively. The plates were incubated for 24 h at 37 °C for bacteria and 48 h at 37 °C for yeast. Growth inhibition was determined by visual observation, which was confirmed with the addition of 30 µL of resazurin (0.005% in PBS; pH 7.2). The MIC was defined as the minimum concentration required to completely inhibit microbial growth. Amphotericin B and ciprofloxacin were used as the antimicrobial standards. The inhibitory effect of DMSO was also tested against the microorganisms. All experiments were performed in triplicate, in at least three independent experiments.

### 2.12. Minimal Bactericidal Concentration (MBC) and Minimal Fungicidal Concentration (MFC)

To determine the MBC and MFC, 10 µL of the suspension obtained from the sub-MIC, MIC, 2× MIC, and 4× MIC concentrations were subcultured onto BHI agar for bacteria or Sabouraud Dextrose agar for yeast. The plates were incubated at 37 °C for 24 h and 48 h, respectively. The microbicidal concentration was determined as the lowest sample concentration in which no growth of microorganisms was observed. From the MIC, the microbicidal effect was considered when the ratio (MBC or MFC/MIC) is ≤4 and microbiostatic when (MBC or MFC/MIC) is >4 [28,29,30].

### 2.13. Sorbitol Assay

The MIC of the EtOAc fraction against *Cryptococcus neoformans* H99 serotype A (ATCC 208821) was determined in RPMI-MOPS medium according to the previously described broth microdilution method in the presence and absence of sorbitol solution (0.8 M) used as a fungal cell wall osmoprotectant. After 48 h of incubation, the MIC was determined, and the values obtained were compared in the presence and absence of sorbitol [31,32].

### 2.14. Galleria mellonella Larvae

The larvae of *G. mellonella* (Greater Wax Moth) used in this study were provided by the Laboratory of Glycobiology of Eukaryotes and kept in sterile Petri dishes at room temperature in the dark. For all experiments, up to 10 healthy larvae weighing 150 and 200 mg were selected for each group. Healthy larvae were considered those that did not show signs of disease, such as gray/dark pigmentation and reduced movements [33].

### 2.15. Toxicity in Galleria mellonella Systemic Model In Vivo

The acute toxic effects of the compounds identified in the extract were evaluated in vivo using the *G. mellonella* larvae model, as previously described [29]. Ten larvae were subjected to 100 mg/Kg of resveratrol or piceatannol. Ten microliters of the standards were injected into the hemocoel of each larva through the last left proleg using a 1 mL insulin syringe. Untouched larvae and larvae injected with saline solution (0.9%, *w*/*v*, NaCl) containing 15% DMSO were used as controls. The larvae were incubated in the dark, at room temperature, in sterile Petri dishes (FirstLab; Pinhais, PR, Brazil) and their survival was monitored at 24 h intervals. Only the larvae displaying no movements upon touch and with high melanization levels were considered dead.

To evaluate the immunological response of *G. mellonella* to stilbene administration, 3 larvae per group were randomly selected after 14 days of incubation, and the hemolymph was extracted by the last left proleg using an insulin syringe (needle 13 mm × 0.45 mm, Descarpack; São Gonçalo, RJ, Brazil). To prevent clotting and melanization, the hemolymph of each larva (10 µL) was transferred into a microcentrifuge tube containing 50 µL of insect physiological saline (IPS; 150 mM NaCl, 5 mM KCl, 100 mM Tris/HCl, 10 mM EDTA, 30 mM sodium citrate, pH 6.9) on ice. After gentle homogenization, 10 μL of the solution was mixed with 10 μL of Trypan blue and the same volume was transferred to a Neubauer chamber for microscopic counting of the number of hemocytes [34,35].

### 2.16. Fungal Infection of Galleria mellonella Larvae

For the infection assay, *C. neoformans* H99 cells were suspended in saline at a concentration of 1 × 10^8^ cells/mL. Using a 1 mL insulin syringe, the larvae were infected through the last left proleg with 10 µL of the cell suspension (1 × 10^6^ cells/larva). Untouched larvae, larvae injected with saline solution (0.9%, *w*/*v*, NaCl) containing 15% DMSO, and infected larvae without treatment were used as controls. After infection, the larvae were incubated in the dark, at room temperature, in sterile Petri dishes for 1 h and then treated with 100 mg/kg of resveratrol or piceatannol. Immediately after treatment, the larvae were incubated again, and the mortality rate was monitored at 24 h intervals post-infection. Death was determined by melanization and lack of movement upon touch [33].

### 2.17. In Silico ADMET Prediction

The in silico profile of absorption, distribution, metabolism, excretion, and toxicity parameters was performed using the ADMET Predictor^TM^ program (Simulations Plus, Inc., Version 7.1, 2014, Lancaster, CA, USA), which consists of estimating the main ADMET pharmacokinetic properties of chemical compounds, based on their molecular structures and structural similarity to compounds with pharmacokinetic and toxicological characteristics previously described in vitro and in vivo, saved in a database [36,37].

### 2.18. Statistical Analysis

The extraction results were expressed as percentages and represented the mean of three independent experiments. In vitro experiments (antimicrobial and antioxidant assays) were performed in triplicate, in at least three independent experiments. In the antioxidant activity tests, all values were expressed as mean values ± standard deviation (SD). Statistical significance between groups was determined by analyses of variance (ANOVA) using Prism^®^ 8.0 software (GraphPad Software, Boston, MA, USA). Differences among means were considered significant at *p* < 0.05 with the use of Tukey’s test.

## 3. Results and Discussion

### 3.1. Extraction Yield and Chemical Analysis

This study employed four solvent systems to extract bioactive compounds from the target material. These solvents, chosen based on their varying polarities, aimed to maximize the yield of diverse phytochemicals. Among the solvents used, the extracts that displayed the highest yields were those obtained through extraction using water and an aqueous mixture with acetone and ethanol, with yields of 28.8%, 23.1%, and 22.1%, respectively. The extraction with pure ethanol was the solvent producing the lowest yield (8.89%). This methodology was intentionally designed as an untargeted approach to broadly recover and profile the diverse range of polar and semi-polar compounds present in the macaúba epicarp.

Afterward, as it displayed promising biological activities (Section 3.3 and Section 3.4), the crude extract using a Me_2_CO:H_2_O mixture was chosen for liquid–liquid extraction with an aqueous mixture of ethyl acetate 1:1 (*v*/*v*), yielding a 13.6% EtOAc fraction and 66.4% of the aqueous fraction. The use of EtOAc, a solvent commonly employed in liquid–liquid extractions for phenol recovery, allowed the selective partitioning of these compounds based on their polarity, ensuring that polyphenols were effectively concentrated into the EtOAc fraction.

The EtOAc fraction underwent adsorption chromatography using medium-pressure liquid chromatography (MPLC). A total of 80 fractions were collected, and those displaying UV absorption at 321 nm (Figure S1) were further analyzed by thin-layer chromatography (TLC). Based on their similar TLC profiles (Figure S2), these fractions were consolidated into two subfractions: subfraction 01 (comprising fractions 12–13) and subfraction 02 (comprising fractions 16–17), with respective yields of 13.4% and 7.35%.

Subfractions 01 and 02 were subjected to UPLC-MS analysis. In subfraction 01, the peaks observed between retention times 28.50 and 30.34 min (Figure S3A) revealed a molecular ion at [M–H]⁻ *m*/*z* = 243 (Figure S3B), along with fragment ions at *m*/*z* 225, 201, 199, 175, and 159 (Figure S3C). This fragmentation pattern is characteristic of the stilbene piceatannol (3,4,3′,5′-tetrahydroxy-trans-stilbene), as supported by previous studies [38,39,40]. The analysis of the peaks with retention time at 18.14 min (Figure S4A) in subfraction 02 revealed a molecular ion at [M–H]^−^ *m*/*z* = 259 (Figure S4B) and product ions with *m*/*z* values of 241 (loss of H_2_O, 18 Da), 217 (loss of C_2_H_2_O, 42 Da), and 175 (losses of two groups of C_2_H_2_O), as the loss of C_2_H_2_O moieties is characteristic from the resorcinol ring of stilbenes [41], suggesting subfraction 02 to be 3,4,5,3′,5′-penta-hydroxy-trans-stilbene, which is also known as PHS [42].

**Figure 1:**
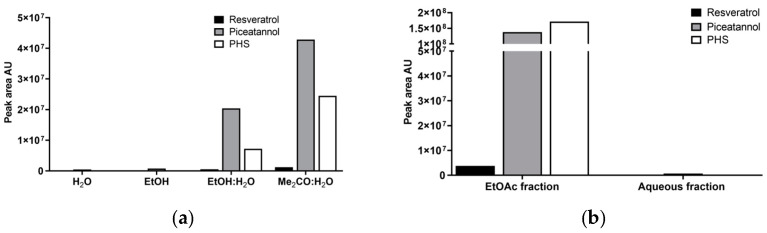
Analysis of the phenolic profile of the crude extracts and fractions of macaúba epicarp: (**a**) effects of different solvents on the extraction of stilbenes; (**b**) analysis of the EtOAc and aqueous fractions obtained from the extract Me_2_CO:H_2_O. The data were reported as the peak areas of the compounds in 20 μg of the dry extract.

The phenolic profile of crude extracts and fractions was analyzed using HPLC-MS, which identified the stilbene resveratrol (3,4′,5-trihydroxy-*trans*-stilbene) through coelution with an analytical standard. Figure 1a illustrates the effectiveness of different solvents in extracting stilbenes from the epicarp of macaúba. The extraction of the epicarp using 100% ethanol or water resulted in lower yields of stilbenes, with the aqueous extract showing no detectable stilbenes and the ethanol extract containing only 17.6 μg of resveratrol per g of the dry extract (1.6 μg of resveratrol per g of epicarp powder) (Table S1). These results are consistent with findings from grape cane studies, which also demonstrated that ethanol alone extracted lower levels of stilbenes than aqueous organic solvents [43]. In contrast, using aqueous organic solvents proved more efficient for extracting stilbenes due to their polarity and solubility properties. Notably, the combination of acetone and water (Me_2_CO:H_2_O) yielded the highest concentrations of these compounds, with 84.0 μg of resveratrol and 1038.9 μg of piceatannol per g of the dry extract (14.9 μg of resveratrol and 184.0 μg of piceatannol per g of epicarp powder), possibly because acetone offered an optimal balance between polarity and hydrophobicity, facilitating better interactions with these bioactive molecules. Stilbene extraction is usually performed with organic solvents such as methanol, ethanol, or aqueous ethanol [44,45,46,47,48]. However, similar studies evaluating the influence of different solvents have been reported for stilbene extraction from other samples [45,49]. Corroborating our results, the extraction of stilbenes from grape canes at 25 °C with 100% ethanol extracted the lowest amounts of stilbene. Furthermore, despite not observing significant differences in ethanol or aqueous methanol use, the authors state that 60% acetone in water extracted the highest amount of stilbenes [49]. In sugarcane after postharvest incubation, the quantification of the stilbenes piceatannol and resveratrol revealed that the maximum concentrations reached 1659 μg/g of dry material for piceatannol and 73 μg/g for resveratrol, which were significantly higher than those observed on macaúba epicarp [50].

The liquid–liquid partition results displayed in Figure 1b showed that only resveratrol exhibited partial solubility in water, with a concentration of 17.5 μg per g of the dry extract (4.1 μg per g of epicarp powder), while the majority of stilbenes preferentially accumulated in the ethyl acetate (EtOAc) fraction. In the EtOAc phase, resveratrol was measured at 229.3 μg and piceatannol at 4645.8 μg per g of the dry extract (15.9 μg and 322.2 μg per g of epicarp powder, respectively). Although PHS was not quantified, its presence was also exclusively observed in the EtOAc fraction. This selective partitioning could be attributed to the moderate hydrophobicity and lower polarity of ethyl acetate, which optimally dissolved stilbenes due to their aromatic and hydrophobic features.

### 3.2. Imaging Mass Spectrometry

**Figure 2:**
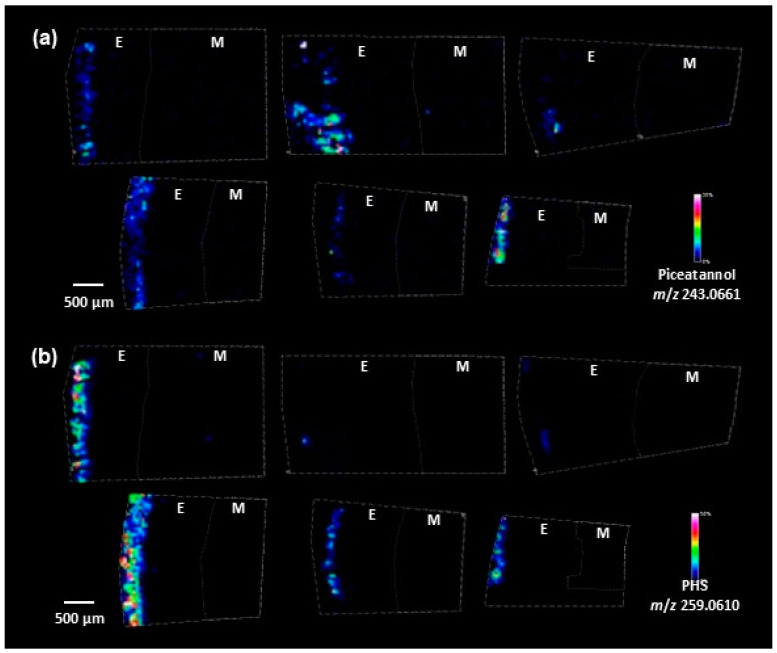
Spatial distribution analysis of stilbenes in macaúba fruit by imaging mass spectrometry in the epicarp (E) and mesocarp (M). (**a**) Piceatannol (*m*/*z* 243.0661) and (**b**) PHS (*m*/*z* 259.0610) were detected exclusively in the external layer of the epicarp across all six fruits analyzed. The color scale indicates ion intensity. Histological images are available on the Supplementary Materials.

To investigate the spatial distribution of stilbenes within macaúba fruit, we performed an imaging mass spectrometry (IMS) analysis on thin sections of the epicarp and mesocarp. The analysis revealed ions with *m*/*z* values of 259.0610 (Figure 2a) and 243.0661 (Figure 2b), corresponding to the M-H ion species of PHS and piceatannol, respectively. These stilbenes were found exclusively in the outer layer of the epicarp (Figure 2), with mass errors of 0.76 ppm and 0.74 ppm, respectively. Interestingly, resveratrol was not detected in any part of the fruit, likely due to its presence in concentrations below the detection threshold or rapid degradation under oxidative conditions. The exclusive localization of PHS and piceatannol in the outer epicarp may be related to the distinct physiological role of this layer, characterized by its darker, brownish appearance and increased hardness and dryness compared to the inner layers (Figure S5A). The protective nature of the epicarp might favor the accumulation of more stable stilbene derivatives, such as PHS and piceatannol, while limiting the stability or biosynthesis of resveratrol. Additionally, due to the hardness of the endocarp, thin sections from the core could not be obtained for analysis.

The incorporation of stilbenes, such as piceatannol, into the lignin structure of macaúba fruit has been reported to contribute significantly to the properties of the endocarp, including enhanced hardness and resilience [10]. A previous study demonstrated that piceatannol and other stilbenes are integrally incorporated into the lignin of palm fruit endocarps via non-canonical biosynthetic pathways participating in the lignification process [51]. Macaúba (*A. aculeata*), carnaúba (*Copernica prunifera*), and coconut (*Cocos nucifera*) contained lignins formed by the common monolignols with stilbenes [51]. In macaúba’s fruit, a difference was detected in the composition of lignin samples taken from macaúba’s epicarp and endocarp, with piceatannol found only in the latter [51].

This incorporation was not observed in the fruit’s epicarp or stalk [10]. Although this finding may contradict our data, the lignin content in the epicarp is less than half of that reported for the endocarp [10]. This difference correlates with the greater stiffness of the endocarp compared to the epicarp, as we observed. Moreover, to our knowledge, no studies have compared stilbene content across different parts of the fruit. Consequently, it is unclear whether the endocarp contains higher levels of piceatannol than the epicarp, which could explain its preferential incorporation into lignin structures in the endocarp.

Additionally, incorporating a monomer into the lignin polymer depends on factors beyond its abundance [52]. Studies in other species, such as *Picea abies*, demonstrate that polyphenolic composition can vary depending on the extraction method and seasonality [53,54]. Furthermore, these variations do not necessarily correlate with the phenolic compounds incorporated into lignin [55]. Further investigations are needed to elucidate the relationship between free stilbene levels and their incorporation into lignin in macaúba fruit tissues.

However, our IMS results confirmed that the stilbenes identified in extraction studies are not due to contamination from the mesocarp, which is in direct contact with the epicarp. In contrast, hexose phosphate, a common sugar metabolite, was detected in both the epicarp and mesocarp (Figure S5B). This metabolite was also identified through LC-MS analysis of the extracts, with MS/MS spectra supporting its identification, though the specific sugar type and glycosidic linkage could not be distinguished.

The application of IMS enabled a detailed assessment of the spatial distribution of these stilbenes within the fruit. The concentration of piceatannol and PHS in the outer layer of the epicarp corroborates the function of stilbenes in plant protection, providing structural fortification, but also imparts antiviral, antibacterial, and antioxidant properties against environmental stress and plant defense [56,57]. Our findings are consistent with previous IMS studies showing the concentration of phenolic compounds in the outer layers of various fruits. However, these prior studies primarily focused on fruits with softer peels or skins, including banana [58], strawberry [59], rabbiteye blueberry [60], and wolfberry [61], which facilitates cryosectioning.

### 3.3. Antioxidant Activity and Protection Against the Oxidative Stress

The antioxidant capacity of the extracts and fractions was initially assessed using the in vitro DPPH test. Table 1 shows the EC50 values for all samples analyzed. The EtOAc fraction exhibited the highest antioxidant capacity with an EC50 of 11.2 μg/mL, followed by the Me_2_CO:H_2_O extract with 37.8 μg/mL. In contrast, the aqueous and ethanolic extracts showed the lowest antioxidant capacities, with EC50 = 495.6 and 470.6 μg/mL, respectively. The standard antioxidant ascorbic acid showed EC50 = 2.5 μg/mL. Regarding the antioxidant capacity, the reference compounds resveratrol and piceatannol displayed results similar to those observed for the Me_2_CO:H_2_O crude extract and the EtOAc fraction.

The stilbenes naturally occurring in *A. aculeata* are known for a wide range of biological activities, including antioxidant and antimicrobial activity [10,62,63,64,65]. The presence of hydroxyl groups has been reported as the main factor responsible for the antioxidant capacity of these phenolic compounds, which can donate electrons [66]. The DPPH free radical scavenging assay is a widely used reproducible method to easily and quickly indicate the preliminary antioxidant capacity of plant extracts [17,23,66]. Furthermore, the interaction of DPPH with an antioxidant potential appears to be related to the number of available hydroxyl groups, suggesting that the DPPH free radical abstracts the phenolic hydrogen from the electron donor molecule [23,67]. Based on this mechanism and the observed results, it is possible that the increased antioxidant activity in the Me_2_CO:H_2_O extract and EtOAc fraction can be linked to the presence of stilbenes found in higher amounts of these extracts.

Oxidative stress caused by the imbalance between the occurrence of free radicals, reactive oxygen/nitrogen species (ROS/RNS), and the body’s antioxidant protective capacity is associated with several pathologies, such as cardiovascular diseases, cancer, aging, and neurodegenerative diseases [68]. Although the human body has a complex endogenous antioxidant defense system formed by antioxidant enzymes, non-enzymatic compounds, and low-molecular-weight scavengers, exogenous sources of antioxidants from the diet are also necessary [68,69]. A recent study demonstrated that stilbenes like resveratrol and piceatannol inhibit superoxide anion generation by xanthine oxidase (XO) through binding to the FAD (flavin adenine dinucleotide) site, leveraging their en-diol structures for potent antioxidant activity [70]. These findings align with the macaúba epicarp’s rich stilbene profile, as expressed in Table 1, reinforcing its potential to mitigate oxidative stress and enhance cellular defenses.

Several plant species, their parts, or even their agro-industrial residues have been reported as considerable sources of bioactive antioxidant compounds, which can be used for a wide range of applications in the food and pharmaceutical industries [17,66,69,71].

**Figure 3:**
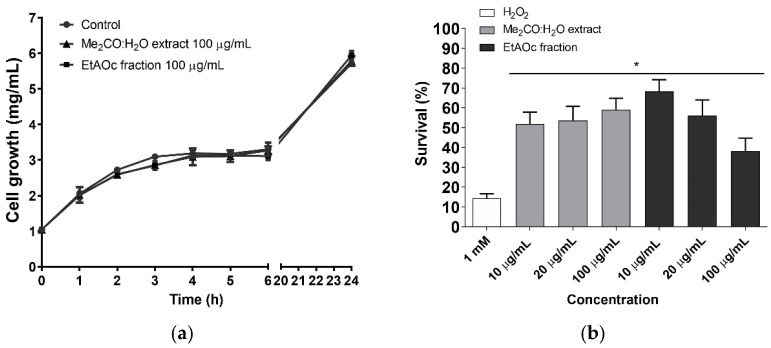
Evaluation of the protective effect against reactive oxygen species. (**a**) Evaluation of cell susceptibility. (**b**) Effect of Me_2_CO:H_2_O extract and EtOAc fraction on survival rates of cells stressed with 1 mM H_2_O_2_. Results are expressed as the survival percentage and represent the mean ± standard deviation of at least three independent experiments. * Represents significantly different results compared to the positive control (1.0 mM H_2_O_2_) at *p* < 0.05.

Thus, aside from DPPH radical scavenging ability, we evaluated the in vivo antioxidant activity of the Me_2_CO:H_2_O crude extract and EtOAc fraction in the *Saccharomyces cerevisiae* model system. Firstly, we investigated whether *S. cerevisiae* cells would be susceptible to the crude extract and fraction. The results shown in Figure 3a demonstrate that the treating cells showed no sign of sensitivity, indicating that the samples were not toxic at the concentration tested.

Regarding the antioxidant potential, it can be observed from Figure 3b that the treatment with the lowest concentration (10 µg/mL) of the Me_2_CO:H_2_O crude extract or EtOAc fraction promoted a significant increase in the survival rates of yeast cells with increases of 3.5-fold (51.7%) and 4.7-fold (68.3%), respectively, against oxidative stress induced by H_2_O_2_ (14.4%). In contrast, non-toxic, higher EtOAc fraction concentrations reduced cellular protection, suggesting stilbenes’ pro-oxidant effects increased oxidative stress [72,73]. Nevertheless, the survival of cells treated with these concentrations remained higher than those directly stressed.

Reports in the literature indicate that phenolic compounds increase antioxidant activity by stimulating the expression of genes encoding endogenous antioxidant enzymes such as glutathione peroxidase, superoxide dismutase, and catalase within the cell, inducing their defense mechanisms to counteract oxidative stress [66,74,75]. However, phenolic compounds such as resveratrol and its metabolites can act as a pro-oxidant, depending on the concentration administered, treatment conditions, the type of cells used, and their interaction with the basal redox state, among other variables [73]. *S. cerevisiae* pretreated with resveratrol demonstrated tolerance to oxidative stress induced by different agents such as hydrogen peroxide, carbon tetrachloride, and cadmium. Furthermore, assays with mutant strains deficient in specific antioxidant defense systems demonstrated that tolerance to H_2_O_2_ and carbon tetrachloride was associated with catalase [63].

### 3.4. Antimicrobial Activity

The antimicrobial potential of macaúba epicarp extracts was assessed using the broth microdilution method against pathogenic microorganisms of both foodborne and medicinal importance. Initial testing of the crude extracts was performed against three bacterial strains (*B. cereus*, *S. aureus*, and MRSA) and two yeast strains (*C. albicans* and *C. neoformans* H99). Among the tested extracts, only the Me_2_CO:H_2_O crude extract demonstrated notable antibacterial and antifungal activity. Table 2 shows the minimal inhibitory concentration (MIC) and minimum bactericidal/fungicidal concentration (MBC/MFC) for the Me_2_CO:H_2_O crude extract, aqueous and EtOAc fractions, as well as the reference compounds resveratrol, and piceatannol.

According to the classification of [76], the Me_2_CO:H_2_O crude extract showed weak antibacterial activity (MIC > 625 μg/mL) against *Bacillus subtilis* (ATCC 6633) with an MIC at 1000 μg/mL and significant antifungal activity (MIC < 100 μg/mL) against different strains of the yeast-like fungus *Cryptococcus neoformans* demonstrating fungicidal activity against *C. neoformans* H99 serotype A (ATCC 208821), *C. neoformans* serotype A (T_1_-444), and *C. neoformans* serotype D (ATCC 24067).

The EtOAc fraction exhibited moderate bactericidal activity against *B. subtilis*, *S*. *aureus*, and MRSA and significant antifungal activity, with a fungicidal effect against *C. neoformans* serotype D and fungistatic activity against *C. neoformans* H99 and *C. neoformans* T1-444. In contrast, the aqueous fraction did not exhibit antimicrobial activity against the microorganisms tested.

Regarding the antimicrobial potential of reference compounds, resveratrol exhibited bacteriostatic activity for *B. subtilis*, *S. aureus*, and MRSA, while piceatannol exhibited bactericidal activity against *B. subtilis* and *S. aureus*, and bacteriostatic activity for the other bacteria. In addition, piceatannol exhibited significant fungicidal activity against all strains of *Cryptococcus*, while resveratrol exhibited inhibitory activity only against *C. neoformans* serotype D (ATCC 24067).

Stilbenes are phytoalexins produced by plants in response to biotic and abiotic stress such as bacteria, fungi, viruses, and ultraviolet radiation, among others. Given this potential, its antimicrobial activity has been investigated against pathogens that are foodborne and of medicinal importance [62,77,78,79]. A previous study analyzed the extract of the spines of *A. totai* against *S. aureus* ATCC 25923, *B. subtilis* ATCC 6623, and *C. albicans* ATCC 10231. As a result, the authors observed moderate bacterial activity for the methanolic extract against *S. aureus* at 500 μg/mL and no activity for the other microorganisms. However, significant activity was observed for both the EtOAc fraction and piceatannol against *S. aureus* at 50 μg/mL and *B. subtilis* at 100 μg/mL, corroborating our results [62].

Negative antifungal results against *Candida* sp. have already been reported. The ethanolic extract of the leaves of *A. aculeata* did not demonstrate antifungal activity when tested against *Candida albicans* 4006 and *Candida parapsilosis* 40038 by the disk diffusion method at concentrations of 25, 50, and 100 mg/mL [44]. Furthermore, a weak antifungal activity of resveratrol was observed against *Candida albicans* at 400 μg/mL, with an inhibition of cell growth lower than 20% [80].

Although the antimicrobial activity of stilbenes has been investigated against several fungi [81], to the best of our knowledge, there are no reports of antifungal activity against *Cryptococcus* species. Our results demonstrate promising antifungal activity of the Me_2_CO:H_2_O extract, EtOAc fraction, and piceatannol against three strains of *C. neoformans*, with CMI ranging from 1.95 to 62.2 μg/mL. Resveratrol only showed activity against *Cryptococcus neoformans* serotype D, with a CMI of 31.2 μg/mL.

Then, to ascertain a possible mechanism of action in the fungal cell, we investigated the involvement of EtOAc fraction on the cell wall synthesis of *C*. *neoformans* H99 serotype A and *C. neoformans* serotype D through a sorbitol assay. However, the MIC of the EtOAc fraction was not increased compared to the MIC obtained without sorbitol (Table 2), suggesting that the stilbenes present in the fraction do not interfere with the fungal cell wall synthesis.

The antimicrobial activity of stilbenes has been associated with different mechanisms of action [78,82]. Antibacterial activity against Gram-negative was related to the destabilization of the outer membrane, interactions with the cytoplasmic membrane, and DNA damage, stimulated by pro-oxidant activity of resveratrol against *Salmonella* Typhimurium [77]. We also observed the membrane damage of *E. coli* mediated by oxidative damage as the primary event [83], and DNA damage as a late event [84]. Furthermore, the stilbene pinosylvin caused the depolarization of the cytoplasmic membrane in *Staphylococcus epidermidis* and *Staphylococcus aureus* (Gram-positives bacteria) [85]. The antifungal activity was already attributed to the downregulation of both the ergosterol [78], peroxidation of the membrane, and the inhibition of the respiration of fungal cells [85]. The pterostilbene was also shown to be capable of causing the destruction of the endoplasmic reticulum, and the nuclear and mitochondrial membranes in the dormant conidia of *Botrytis cinerea* [81].

#### Influence of Resveratrol and Piceatannol on Infection of Galleria mellonella by Cryptococcus Neoformans

Finally, the antifungal potential of the stilbenes was investigated in the *Galleria mellonella* model infected with *C. neoformans* H99. Firstly, toxicity assays were carried out to evaluate if the compounds adversely affected the larvae when subjected to a dose of 100 mg/Kg injected through the last left proleg. The results demonstrated that all animals survived and their life cycle was not affected. After 14 days of exposure to the stilbenes, hemolymph analysis indicated an increased hemocyte density by 53.5% (resveratrol) and 169.4% (piceatannol) when compared with the control (saline solution containing 15% DMSO).

**Figure 4:**
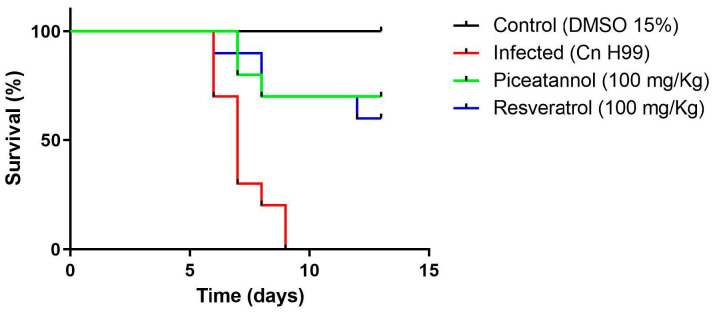
Evaluation of the antifungal potential of stilbenes in *Galleria mellonella* larvae infected with *C. neoformans* H99. Larvae infected with 1 × 10^6^ cells/larva were incubated in the dark, at room temperature, in sterilized Petri dishes for 1 h. They were then treated with 100 mg/kg of resveratrol or piceatannol.

Regarding the antifungal potential, we observed in Figure 4 that all larvae infected with *C. neoformans* (1 × 10^6^ cells/larva) died until the 9th day of injection. Treatment of larvae with 100 mg/kg of resveratrol or piceatannol increased the survival rate to 60% and 70%, respectively, on the 13th day of monitoring.

The use of *Galleria mellonella* (Greater Wax Moth) larvae in in vivo studies has become quite useful because it is a simple model, easy to maintain, and does not require approval from the ethics committee for animal experimentation because it is an invertebrate [34]. The similarity between the innate immune systems of larvae and humans allows their use in studies to determine toxicity, efficacy of new antimicrobial compounds, immune response, and evaluation of virulence factors [33,34,86].

Although trans-resveratrol did not demonstrate activity against *C. neoformans* H99 in the in vitro test, larvae treated with this stilbene demonstrated good survival against infection compared to untreated larvae. This phenomenon may be associated with the fact that resveratrol induces an increase in the density of hemocytes, contributing to the control of fungal proliferation, since hemocytes are responsible for the production of many humoral molecules that favor your immune system [86].

A recent study evaluated the antifungal activity of a rod-shaped gold nanoparticle containing the stilbene resveratrol in a *Galleria mellonella* model, which exhibited significant antifungal activity against *C. albicans* (2.46 µg/mL). At concentrations up to 20x the MIC value (49.2 µg/mL), larvae maintained 100% survival, indicating the potential of this nanoparticle to treat *C. albicans* infections [87].

### 3.5. In Silico ADMET Prediction

After evaluating the antimicrobial activity of the isolated compounds, ADMET (absorption, distribution, metabolism, excretion, and toxicity) modeling can provide a comprehensive prediction of the pharmacokinetic and safety profiles of potentially therapeutic compounds, such as the stilbenes studied, into preclinical or clinical stages. This analysis can ensure that promising antimicrobial agents, like resveratrol and piceatannol derived from the macaúba epicarp, demonstrate potent activity while possessing favorable pharmacological and safety characteristics, thus enhancing their applicability in pharmaceutical development.

The in silico predictive analysis of physicochemical descriptors—lipophilicity, water solubility, and toxicity (Table S2)—for PHS, piceatannol, resveratrol, and amphotericin B reveals important insights into their drug-like properties. PHS, piceatannol, and resveratrol meet Lipinski’s Rule of Five criteria, each having molecular weights under 300 g/mol and suitable hydrogen bond parameters, indicating favorable absorption and permeability for oral bioavailability [88]. Although amphotericin B has a high molecular weight, formulation strategies like liposomal encapsulation improve its bioavailability and reduce systemic toxicity [89,90]. The lower molecular weights of PHS, piceatannol, and resveratrol enhance their potential for effective oral administration. All compounds exhibit moderate lipophilicity with log Po/w values under three, which is crucial for drug distribution [88,91]. Resveratrol’s topological polar surface area (TPSA) is 60.69 Å^2^, close to the optimal threshold for oral absorption, while PHS (101.15 Å^2^) and piceatannol (87.92 Å^2^) may face challenges for absorption. Amphotericin B’s higher TPSA limits its permeability; thus, it is used via non-oral delivery methods [37,90,92].

These TPSA differences suggest that resveratrol is the most suitable for oral absorption, while PHS and piceatannol may need specialized delivery systems. Toxicity predictions show low toxicity for PHS, piceatannol, and resveratrol, congruent with their natural origins [93,94]. Resveratrol is well tolerated in various studies due to its antioxidative and anti-inflammatory effects [95]. Piceatannol, a resveratrol metabolite, shares these properties with low toxicity [74,95]. In contrast, amphotericin B has significant nephrotoxicity, limiting its therapeutic use [90] and necessitating liposomal formulations to reduce toxicity while maintaining antifungal efficacy [89,90]. Thus, while amphotericin B’s toxicity constrains its utility, PHS, piceatannol, and resveratrol have favorable toxicity profiles for further pharmacological development. A clinical study indicates resveratrol’s cardiovascular benefits and endothelial function improvement, though its bioavailability is limited by rapid metabolism [96]. The TPSA of 60.69 Å^2^ aligns with clinical observations of efficient absorption but reduced systemic availability. Beneficial effects at various doses suggest that therapeutic outcomes are achievable despite limited bioavailability, particularly at higher doses [96]. Piceatannol, sharing resveratrol’s pharmacological properties, shows biological activity, but the study does not address its pharmacokinetics [74,97]. Its TPSA of 87.92 Å^2^ may limit passive absorption; however, the observed biological effects suggest that therapeutic concentrations can still be achieved despite potential limitations [97].

## 4. Conclusions

In conclusion, this study underscores the significant potential of *A. aculeata* epicarp extracts as a sustainable source of bioactive stilbenes, such as piceatannol, PHS, and resveratrol, possessing noteworthy biological properties. Our comprehensive characterization used advanced analytical techniques, including imaging mass spectrometry (IMS) and high-performance liquid chromatography coupled with mass spectrometry (HPLC-MS), facilitating the identification and mapping of these compounds within the epicarp. Also, we showcased the efficacy of IMS in elucidating the complex chemical compositions found in plant matrices.

The extracts demonstrated a robust antioxidant capacity, effectively reducing oxidative stress in *Saccharomyces cerevisiae* cells, which highlights their potential to mitigate oxidative damage in biological systems. Moreover, the significant antifungal activity observed against *Cryptococcus neoformans* and the increased survival rates of *Galleria mellonella* larvae that are infected with this pathogen position these stilbenes as promising therapeutic agents in the fight against fungal infections. The in silico ADMET analyses have been instrumental in establishing a safety profile for piceatannol, PHS, and resveratrol, revealing low toxicity and supporting their viability for pharmaceutical development. Careful consideration of the pro-oxidant effects observed at higher concentrations offers critical insights into the redox properties of stilbenes, illuminating the need for future research into the dose-dependent relationships and mechanisms underlying these effects.

This work not only fills substantial gaps of the chemical and biological potential of macaúba epicarp but also advocates for a sustainable approach in repurposing agricultural by-products that are frequently overlooked. Future studies should delve deeper into the specific mechanisms driving the bioactivities of these stilbenes, including their interactions at the molecular level, as well as strategies for industrial-scale applications. Such investigations will be pivotal in advancing the utilization of agricultural residues in high-value solutions, ultimately contributing to improved sustainability and innovation within the food and pharmaceutical industries.

## Figures and Tables

**Table 1:** DPPH free radical scavenging of macaúba epicarp extracts/fractions and stilbenes (Sigma-Aldrich^®^).

Crude Extract/Fraction/Compound	Antioxidant Activity	
	(EC50—μg/mL)	(EC50—mg/g Dried Epicarp)
H_2_O	495.6 ± 17.9 ^a^	14.27 ± 0.51 ^a^
EtOH	470.6 ± 31.3 ^a^	4.18 ± 0.27 ^b^
EtOH:H_2_O	144.5 ± 7.0 ^b^	3.18 ± 0.15 ^c^
Me_2_CO:H_2_O	37.8 ± 2.4 ^c^	0.66 ± 0.04 ^d^
EtOAc fraction *	11.2 ± 3.8 ^c^	0.026 ± 0.01 ^d,e^
Aqueous fraction *	134.8 ± 5.1 ^b^	1.57 ± 0.06 ^d,f^
Resveratrol	12.8 ± 0.5 ^c^	-
Piceatannol	8.58 ± 0.3 ^c^	-
Acid ascorbic	2.5 ± 0.1 ^c^	-

Results expressed as mean ± standard deviation (n = 3). Different superscript letters represent statistically significant differences (*p* < 0.05) among samples. * Fractions of the liquid–liquid partition with ethyl acetate (1:1) of the extract Me_2_CO:H_2_O.

**Table 2:** Minimum inhibitory concentration and minimum bactericidal/fungicidal concentration of macaúba epicarp extracts/fractions and stilbenes (Sigma-Aldrich^®^).

Microorganism	Me_2_CO:H_2_O	EtOAc Fraction	Aqueous Fraction	Resveratrol	Piceatannol	Cipro	AMB
	CMI/CMB or CMF						
*S. enterica*	nd	nd	nd	nd	250/nd	-	-
*B. subtilis*	1000/1000	125/250	nd	125/125	125/125	-	-
*S. aureus*	nd	125/500	nd	62.5/nd	62.5/125	0.2	-
MRSA	nd	250/1000	nd	31.2/nd	125/nd	12.5	-
*C. albicans*	nd	nd	nd	nd	nd	-	0.02
*C. neoformans*	62.2/500	7.8/7.8	nd	31.2/62.5	1.95/1.95	-	0.01
*C. neoformans* H99	31.2/125	31.2/250	nd	nd	31.2/62.5	-	0.01
*C. neoformans* T_1_-444	31.2/125	15.6/250	nd	nd	15.6/62.5	-	0.05

Results expressed in μg/mL; nd—not detected at the maximum concentration tested; CMI—minimal inhibitory concentration; CMB—minimal bactericidal concentration; CMF—minimal fungicidal concentration; EtOAc—ethyl acetate fraction; Cipro—ciprofloxacin; AMB—amphotericin B; *S. enterica* = *Salmonella enterica* serovar Typhimurium (ATCC 14028); *B. subtilis* = *Bacillus subtilis* (ATCC 6633); *S. aureus* = *Staphylococcus aureus* (ATCC 6538); MRSA = methicillin-resistant *Staphylococcus aureus* (BMB 9393); *C. albicans* = *Candida albicans* serotype B (ATCC 36802); *C. neoformans* = *Cryptococcus neoformans* serotype D (ATCC 24067); *C. neoformans* H99 = *Cryptococcus neoformans* H99 serotype A (ATCC 208821); and *C. neoformans* T1 444 = *Cryptococcus neoformans* serotype A (T_1_-444).

## Data Availability

All data sources used in this study can be available upon a reasonable request to the corresponding author.

## Supplementary Materials

The following supporting information can be downloaded at https://www.mdpi.com/article/10.3390/antiox14020181/s1, Figure S1: MPLC Chromatogram of the EtOAc fraction (Me_2_CO:H_2_O) from macaúba epicarp. Gradient: 10–86% B in 11 min; 86% B until 16 min. Flow rate: 75 mL/min. UV: 280/321 nm. Selected fractions (321 nm) for TLC. Figure S2: TLC of MPLC fractions (acetone/toluene/formic acid 3:3:0.1). UV (365 nm): subfraction 01 (fractions 12–13), subfraction 02 (fractions 16–17). Figure S3: UPLC-MS of subfraction 01. Gradient: 5–90% B in 41 min; flow: 0.3 mL/min. (A) Chromatogram; (B) mass spectrum (*m*/*z* 50–500); (C) ion *m*/*z* 243 (fragments: *m*/*z* 225, 201, 199, 175, 159). Figure S4: UPLC-MS of subfraction 02. Gradient: 5–90% B in 41 min; flow: 0.3 mL/min. (A) Chromatogram; (B) mass spectrum (*m*/*z* 50–500); (C) ion *m*/*z* 259 (fragments: *m*/*z* 241, 217, 175). Table S1: Quantification of piceatannol/resveratrol in crude extracts and fractions. Figure S5: IMS of stilbenes in macaúba epicarp and mesocarp. (a) Histological image; (b) hexose phosphate (*m*/*z* 259.0223). Table S2: In silico ADMET: physicochemical properties, lipophilicity, and solubility of PHS, piceatannol, resveratrol, and amphotericin B.
