# Supplementary material for: Exploring the Epicarp Potential from Acrocomia aculeata Fruits: Chemical Analysis, Antioxidant and Antimicrobial Activities
Source: Antioxidants (Basel). 2025 Feb 4;14(2):181. doi: 10.3390/antiox14020181 (PMC11852208; doi:10.3390/antiox14020181)
Supplement: Supplementary file 1 [file antioxidants-14-00181-s001.zip › antioxidants-3401614-supplementary.pdf]

Supplementary material

Exploring the Epicarp Potential from *Acrocomia aculeata* Fruits: Chemical Analysis, Antioxidant and Antimicrobial Activities

Fabiane da Conceição Vieira Santos [1], Gabriel Rocha Martins [2], Sandra Regina da Silva Luiz [3], Isadora de Araújo Oliveira [4], Leandro Pereira da Silva [5], Antonio Jorge Ribeiro da Silva [6], Marcos Dias Pereira [7], Rosana Conrado Lopes [8], Celuta Sales Alviano [9,†] and Daniela Sales Alviano Moreno [9,*]

[1] Graduate Program in Food Science (PPGCAL), Institute of Chemistry (IQ), Federal University of Rio de Janeiro (UFRJ), Cidade Universitária, Rio de Janeiro, RJ 21941-909, Brazil; fabiiane@ufrj.br
[2] Department of Pharmacy, School of Pharmaceutical Sciences, University of São Paulo, São Paulo, SP, 05508-000, Brazil.; gabrielrmartins@usp.br
[3] Graduate Program in Science (PPG-Micro), Department of General Microbiology, Institute of Microbiology Paulo de Góes (IMPG), Federal University of Rio de Janeiro (UFRJ), Cidade Universitária, Rio de Janeiro, RJ 21941-902, Brazil; sandraregipn2514@micro.ufrj.br
[4] Institute of Biophysics Carlos Chagas Filho, Centro de Espectrometria de Massas de Biomoléculas (CEMBIO), Federal University of Rio de Janeiro (UFRJ), Cidade Universitária, Rio de Janeiro, RJ 21941-902, Brazil; isadora@biof.ufrj.br
[5] Graduate Program in Plant Biotechnology and Bioprocesses (PBV), Federal University of Rio de Janeiro (UFRJ), Cidade Universitária , Rio de Janeiro, RJ 21941-902, Brazil; leandropereiira@ufrj.br
[6] Natural Products Research Institute (IPPN), Federal University of Rio de Janeiro (UFRJ), Cidade Universitária, Rio de Janeiro, RJ 21941-902, Brazil; ajorge@ippn.ufrj.br
[7] Department of Biochemistry, Institute of Chemistry (IQ), Federal University of Rio de Janeiro (UFRJ), Cidade Universitária, Rio de Janeiro, RJ 21941-909, Brazil; marcosdp@iq.ufrj.br
[8] Department of Botany, Institute of Biology (IB), Federal University of Rio de Janeiro (UFRJ), Cidade Universitária, Rio de Janeiro, RJ 21941-909, Brazil; rosana@biologia.ufrj.br
[9] Department of General Microbiology, Institute of Microbiology Paulo de Góes (IMPG), Federal University of Rio de Janeiro (UFRJ), Cidade Universitária, Rio de Janeiro, RJ 21941-902, Brazil; alviano@micro.ufrj.br
* Correspondence: danialviano@micro.ufrj.br

† *in memoriam*

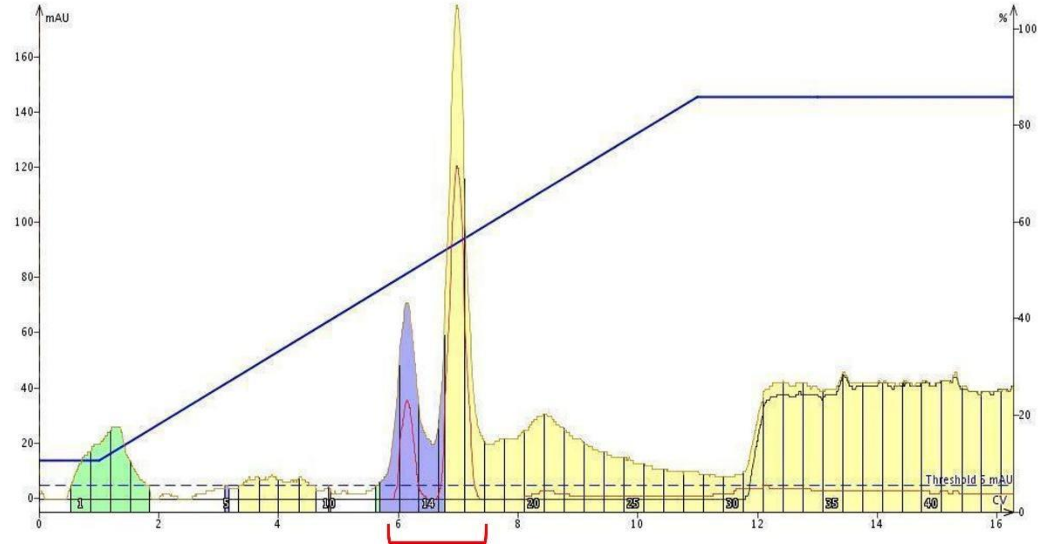

Figure S1. MPLC Chromatogram of the ethyl acetate fraction of the Me_2CO:H_2O extract of macaúba epicarp. The mobile phase was a binary gradient of toluene (A) and acetone (B). The elution profile was 0–1 min 10% B; 1–11 min 10-86% B; 11–16 min 86% B. The flow rate was 75 mL/min, the detector wavelength was 280 and 321 nm, and the injection volume was 15 mL (2,8 mg/mL). The outer signage (red) represents the fractions with UV absorption at 321 nm selected for thin layer chromatography (TLC) analysis.

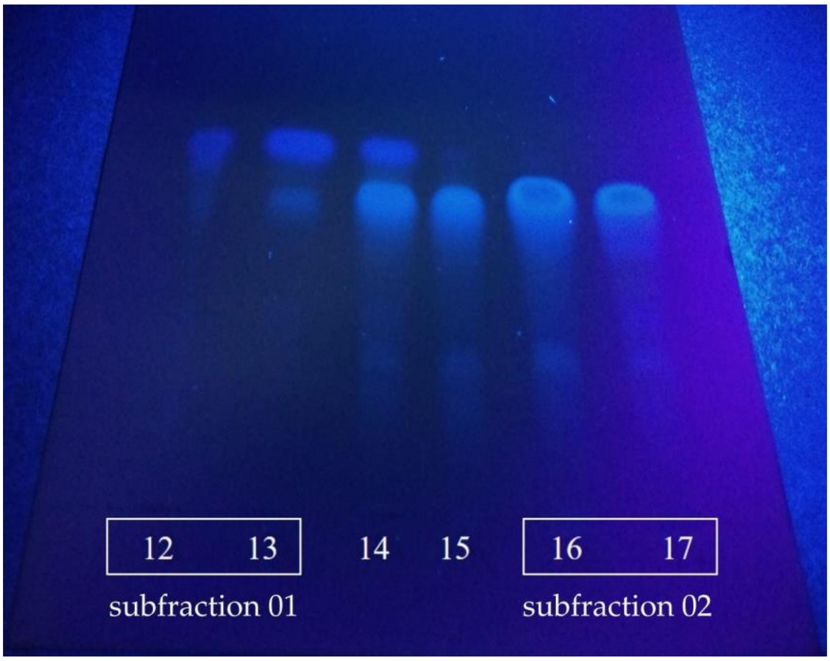

Figure S2. Thin layer chromatography of fractions collected by MPLC. Analysis performed on silica gel 60 F254 plates (Merck), with acetone/toluene/formic acid (3:3:0.1, v/v/v) as mobile phase. The plate was analyzed under UV light (365 nm), and regrouped by the similarity of the chromatographic profile, giving rise to subfraction 01 (fractions 12–13) and subfraction 02 (fractions 16–17).

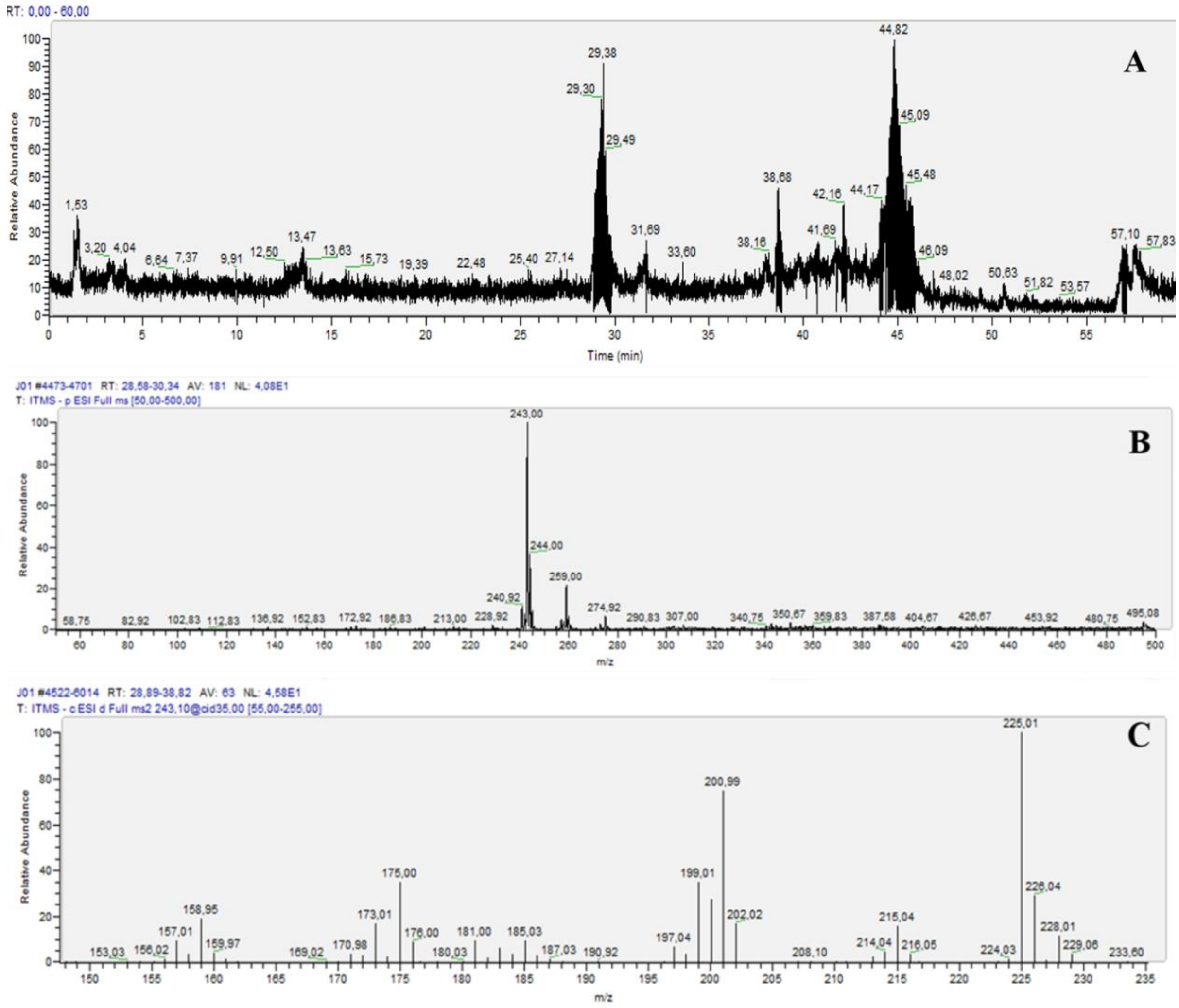

Figure S3. UPLC-MS Chromatograms of the subfraction 01 obtained from the EtOAc fraction of the $Me_2CO:H_2O$ extract of macaúba epicarp. Elution was performed at 0.3 mL/min in a gradient of mobile phases of water (A) and acetonitrile (B), both containing 0.1% formic acid, as follows: 0 min 5% B, 2 min 5% B, 30 min 15% B, 40 min 30% B, 41 min 90% B, 52 min 90% B, 53 min 5% B, 60 min 5% B. The flow rate was 0.30 mL/min. (A) Chromatogram. (B) Mass spectrum acquired in full-scan mode, covering a mass-to-charge ratio (*m*/*z*) range of 50–500. (C) Fragmentation of the molecular ion *m*/*z* = 243 (fragment ions at *m*/*z* 225, 201, 199, 175, and 159)

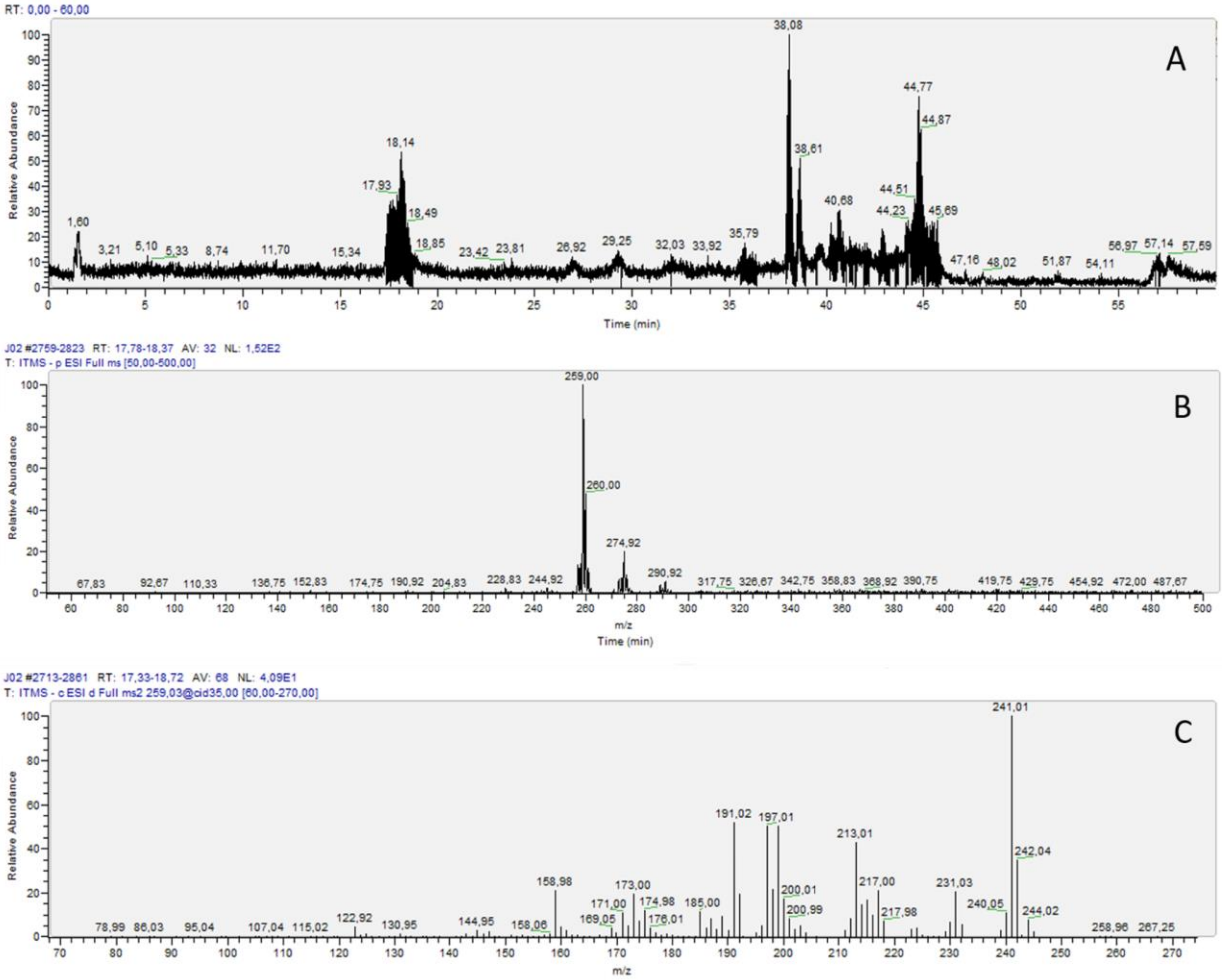

Figure S4. UPLC-MS Chromatograms of the subfraction 02 obtained from the EtOAc fraction of the Me_2CO:H_2O extract of macaúba epicarp. Elution was performed at 0.3 mL/min in a gradient of mobile phases of water (A) and acetonitrile (B), both containing 0.1% formic acid, as follows: 0 min 5% B, 2 min 5% B, 30 min 15% B, 40 min 30% B, 41 min 90% B, 52 min 90% B, 53 min 5% B, 60 min 5% B. The flow rate was 0.30 mL/min. (A) Chromatogram. (B) Mass spectrum acquired in full-scan mode, covering a mass-to-charge ratio (m/z) range of 50–500. (C) Fragmentation of the molecular ion m/z = 259 (fragment ions at m/z 241, 217 and 175).

Table S1. Quantification of piceatannol and resveratrol in samples of the crude extracts and fractions.

	Per dry extract (g)		Per epicarp powder (g)	
Extraction	**Resveratrol (µg)**	**Piceatannol (µg)**	**Resveratrol (µg)**	**Piceatannol (µg)**
H_2O	N.D.	N.D.	N.D.	N.D.
EtOH	17.60	N.D.	1.56	N.D.
EtOH:H_2O	45.43	187.92	10.02	41.48
Me_2CO:H_2O	84.02	1038.8	14.88	184.0
EtOAc fraction *	229.3	4645.8	15.9	322.2
Aqueous fraction *	17.5	N.D.	4.11	N.D.

The analysis was performed by LC-MS (Shimadzu, Japan). Elution was performed at 0.3 mL/min in a gradient of mobile phases of water (A) and acetonitrile (B), both containing 0.1% formic acid, as follows: 0 min 5% B, 2 min 5% B, 30 min 15% B, 40 min 30% B, 41 min 90% B, 52 min 90% B, 53 min 5% B, 60 min 5% B. The quantification was performed by analyzing calibration curves of a mixture of samples added with 0.625 – 20 µM (resveratrol) and 1.25 – 40 µM (piceatannol) of commercial standards of these molecules. The concentration of the compounds was calculated using their respective standard curves: resveratrol, y = 5.0163x10-7x + 0.13560; R2 = 0.99017; piceatannol, y = 3.1084x10-7x - 4.8065; R2 = 0.94928. Considering the extraction yield, the values found were converted to µg/g dry extract and µg/g epicarp powder. ND – Not detected; H_2O – water; EtOH – ethanol; Me_2CO – acetone; EtOAc – ethyl acetate; *Fractions of the liquid-liquid partition with ethyl acetate (1:1) of the extract Me_2CO:H_2O.

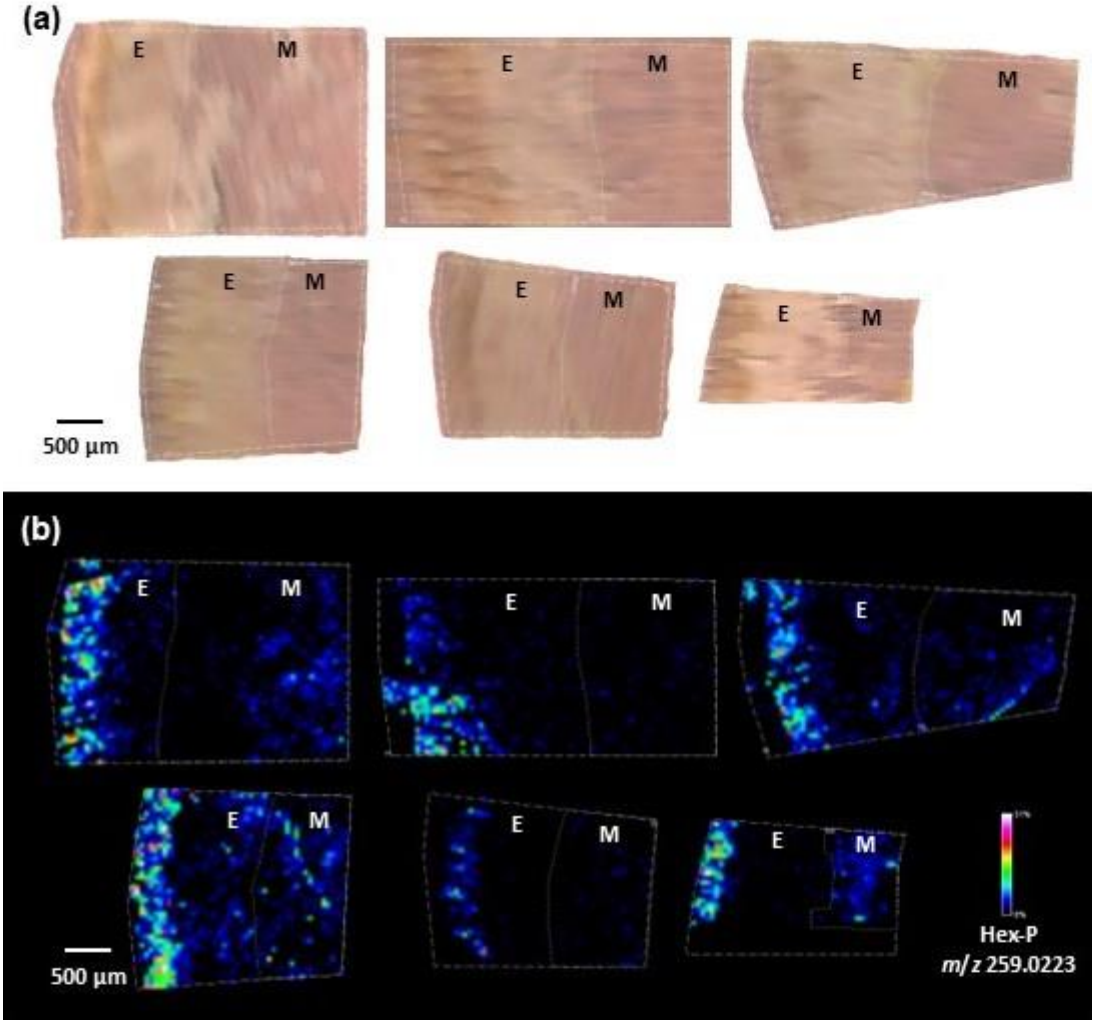

Figure S5. Spatial distribution analysis of stilbenes in macaúba fruit by imaging mass spectrometry in the epicarp (E) and mesocarp (M). (**a**) histological image and (**b**) hexose phosphate (*m/z* 259.0223) detected in the epicarp and mesocarp across all six fruits analyzed. The color scale indicates ion intensity. The color scale indicates ion intensity.

Table S2. *In silico* predictive results of physicochemical descriptors, lipophilicity, and water solubility of PHS, Piceatannol, Resveratrol, Amphotericin B compounds.

	PHS	Piceatannol	Resveratrol	Amphotericin B
**Physicochemical properties**				
Molecular weight	260.24 g/mol	244.24 g/mol	228.24 g/mol	924.08 g/mol
Nº rotatable bonds	2	2	2	3
Nº H-bond acceptors	5	4	3	18
Nº H-bond donors	5	4	3	12
TPSA	101.15 Å^2	80.92 Å^2	60.69 Å^2	319.61 Å^2
**Lipophilicity**				
Log Po/w (iLOGP)	0.98	1.61	1.71	3.76
Log Po/w (XLOGP3)	2.42	2.86	3.13	0.00
Log Po/w (WLOGP)	2.17	2.46	2.76	0.71
Log Po/w (MLOGP)	1.11	1.67	2.26	- 1.74
Log Po/w (SILICOS-IT)	1.60	2.08	2.57	-4.66
Consensus Log Po/w	1.66	2.14	2.48	-0.39
**Water Solubility**				
Log S (ESOL)	-3.31	-3.52	-3.62	-5.37
Class	Soluble	Soluble	Soluble	Moderately soluble

The *in silico* profile of the main pharmacokinetic properties of chemical compounds was performed using the ADMET Predictor™ program (Simulations Plus, Inc - Version 7.1, 2014 - Lancaster, California).
